# Assessing patients’ knowledge immediately after obtaining the antibacterial drug azithromycin: a community pharmacy-based cross-sectional study

**DOI:** 10.1186/s13104-025-07447-1

**Published:** 2025-08-18

**Authors:** Asmaa S. Mohamed, Hosam M. A. Refaei, Remon R. Rofaeil

**Affiliations:** 1 Clinical Pharmacy and Pharmacy Practice Department, Faculty of Pharmacy, Port Said University, Port Said, Egypt; 2 Clinical Pharmacy and Pharmacy Practice Department, Faculty of Pharmacy, East Port Said National University, Port Said, Egypt; 3 Internal Medicine and Biochemistry Departments, Egypt Ministry of Health and Population, Directorate of Health Affairs, Minia, Egypt; 4 Pharmacology Department, Faculty of Pharmacy, Deraya University, Minia, Egypt; 5 Pharmacology Department, Faculty of Medicine, of Clinical Pharmacy and Pharmacy Practice, Faculty of Pharmacy, Minia University, Port said University, Minia, Egypt

**Keywords:** Medication knowledge, Azithromycin, Antibacterial drug misuse, Self-medication

## Abstract

**Objective:**

This cross-sectional study was conducted across 38 community pharmacies and included 418 patients who were dispensed oral azithromycin. Pharmacists administered a structured questionnaire to collect demographic information and assess patients’ knowledge regarding azithromycin use. Knowledge was evaluated based on responses related to the medication’s name, dosage, frequency, potential side effects, and intended purpose. Based on their scores, participants were categorized as having poor (< 50%), average (50–70%), or good (> 70%) knowledge. The aim of this study was to assess patients’ knowledge of azithromycin use and identify factors influencing their level of understanding.

**Results:**

A total of 418 participants completed the questionnaire, with a mean age of 35.27 years; 75.60% were under 40 years old, and 62.20% were female. The majority were married (81.82%), resided in urban areas (57.89%), and held a university degree (67.70%). Azithromycin was prescribed by physicians for 62.20% of participants, while 29.67% obtained it directly from pharmacists via over-the-counter (OTC) access, and 8.13% self-medicated. Knowledge scores differed significantly according to marital status, place of residence, education level, and socioeconomic status, with higher scores observed among married, urban, university-educated, and higher-income individuals (*p* < 0.05). In contrast, age, gender, smoking status, and diabetes mellitus showed no significant association with knowledge scores. Overall, 21.53% of participants had poor knowledge, 46.41% had average knowledge, and 32.06% demonstrated good knowledge.

**Supplementary Information:**

The online version contains supplementary material available at 10.1186/s13104-025-07447-1.

## Introduction

Antimicrobial resistance (AMR) is a major public health issue, making bacterial infections more difficult to treat, increasing healthcare costs, prolonging illness, and raising mortality rates [1, 2]. Misuse and overprescription of antibiotics, especially for viral infections mistaken as bacterial, significantly drive AMR [3, 4].

**Fig. 1:**
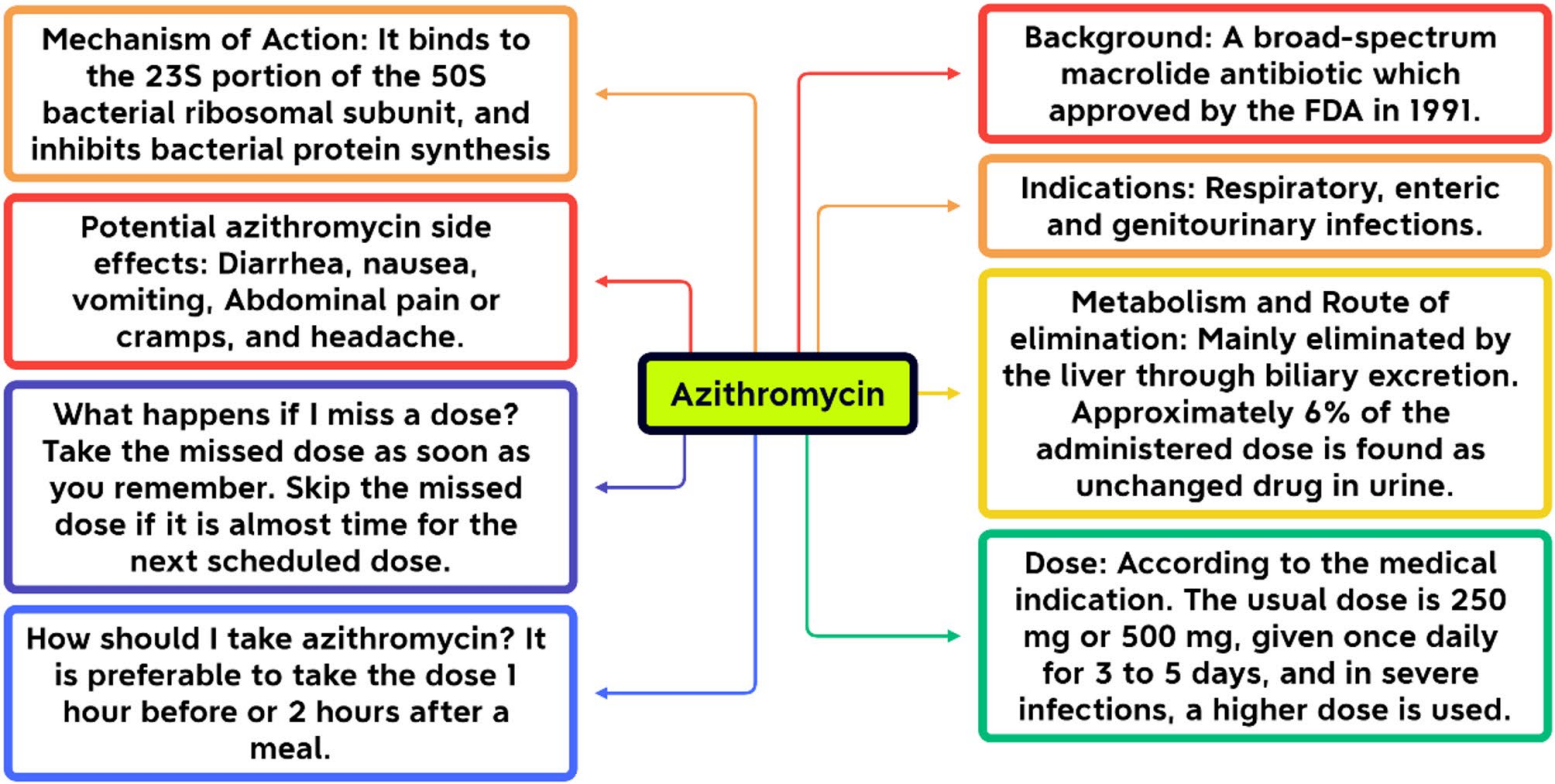
Overview of azithromycin

Azithromycin, a widely used macrolide antibiotic, treats respiratory, sexually transmitted, and skin infections due to its broad-spectrum activity, once-daily dosing, and short treatment duration [5, 6]. However, its overuse, often for inappropriate conditions, exacerbates antimicrobial resistance. Compared to other macrolides, it has a lower risk of cardiac side effects like QT interval prolongation, contributing to its popularity [6–8]. An overview of azithromycin is presented in Fig. 1 [9–11].

Azithromycin, typically prescription-only, is sometimes available over-the-counter in some pharmacies. Its frequent prescriptions, self-medication trends, and increased public attention during health crises like COVID-19 make it a key subject for studying public understanding of antibiotics. This study aims to evaluate patients’ understanding of azithromycin use and to identify factors influencing their knowledge.

## Methods

### Study design and setting

This cross-sectional study took place in 38 community pharmacies in Port Said, Egypt, a region with urban and rural areas near the Suez Canal, ideal for examining public health behaviors and pharmacy access. Pharmacies were chosen using stratified sampling to represent urban (28) and rural (10) areas proportionally. Four pharmacies declined participation due to time or other constraints, but the rest agreed.

### Study population

**Fig. 2:**
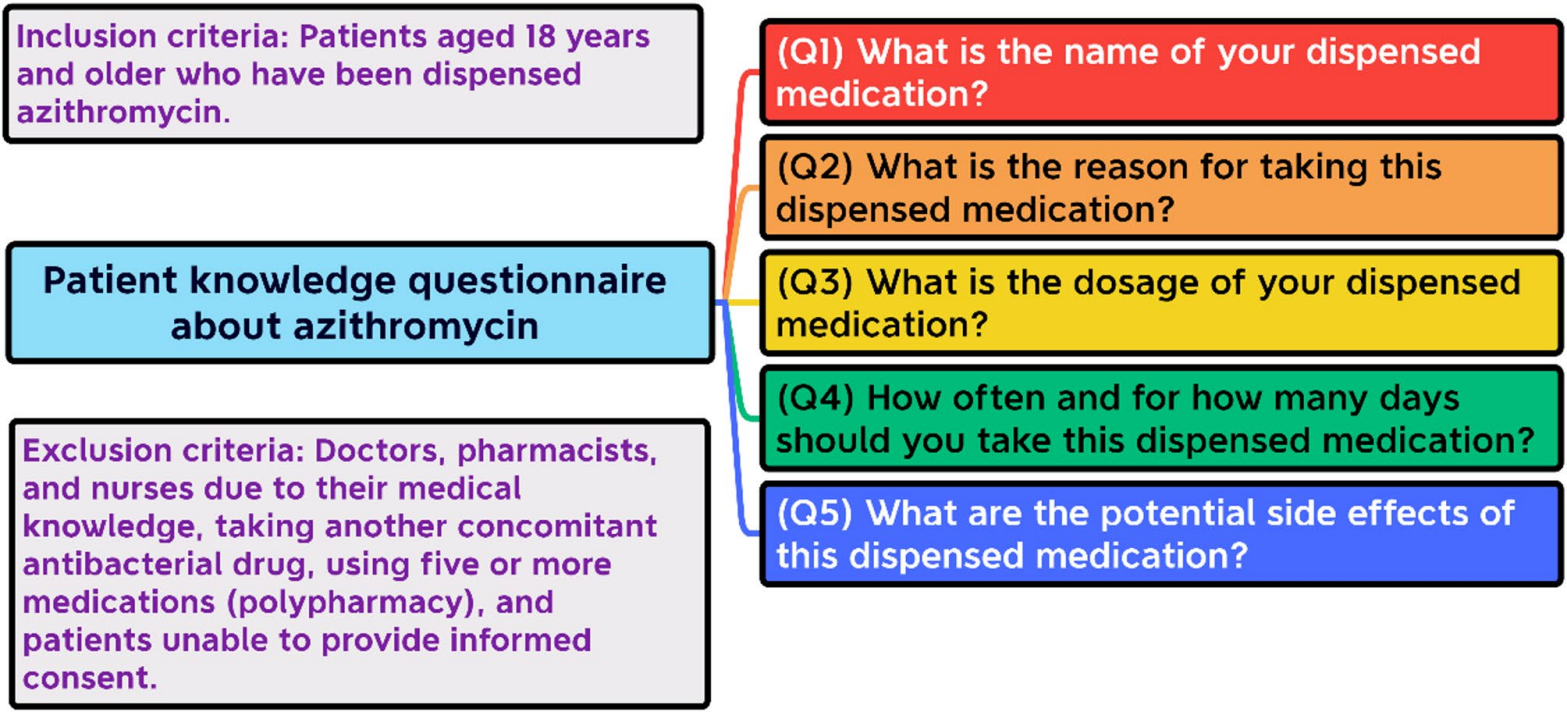
The main study steps

The study enrolled 418 participants, excluding those on multiple medications or antibiotics to focus on azithromycin use (see Fig. 2). Data was collected via structured face-to-face interviews by trained pharmacists using a standardized questionnaire to gather demographic information and assess patient knowledge. Medical professionals, such as doctors, pharmacists, and nurses, were excluded to avoid bias from their advanced pharmacological expertise.

#### Ethics approval

This study was conducted following approval from the Port-Said University Research Ethics Committee.

#### Sampling method

The sample population was determined using a consecutive sampling method. The minimal sample size was calculated using the formula: *n* = Z^2^*P*(1-P)/E^2^, (n) = the required sample size, Z = the critical value for the confidence level (for 95% confidence, Z = 1.96), *P* = the prevalence of patients with insufficient knowledge about their medications (estimated at 50% to maximize sample size, i.e., 0.5), E is the margin of error (in this study, 0.05). Thus, the minimum required sample size was calculated to be 384 participants. To account for potential non-response or incomplete data, the sample size was increased to 418 participants.

### Data collection tool and outcomes

The questionnaire consisted of five questions designed to assess key information about the drug, including its name, intended use, daily dosage, frequency of administration, and potential side effects (see Fig. 2). The full English version of the questionnaire is provided as a supplementary file for reference.

Participants were recruited immediately after obtaining azithromycin, regardless of whether it was prescribed by a physician, dispensed OTC, or obtained for self-medication. Pharmacists administered the questionnaire in a private area of the pharmacy to minimize external influence. Importantly, the pharmacists conducting the interviews were not employed by the participating pharmacies; rather, they were trained data collectors recruited specifically for this study. The roles of prescriber and dispenser were clearly distinguished: physicians prescribed azithromycin, and community pharmacists dispensed the medication.

The questionnaire consisted of five core questions, and participants who answered all of them correctly received a patient knowledge score (PKS) of 100%. Each question contributed 20% to the total score. A correct answer earned 20%, a partially correct response earned 10%, and an incorrect or “don’t know” response received 0%. For example, answers to the question about the name of the medication were scored as 20% for a comprehensive and accurate response, 10% for a partially correct or incomplete answer, and 0% for incorrect or “don’t know” response. An exception was applied to the final question, which assessed knowledge of azithromycin’s potential side effects and was scored differently. Each correct identification of a side effect was awarded 5% points, with four required for a maximum of 20%.

In the assessment, only potential adverse effects that are documented in clinical references and frequently included in patient information leaflets, such as some gastrointestinal disturbances, allergic reactions, and headache, were considered correct. Rarely reported or less substantiated side effects, including palpitations, fatigue, and dizziness, were marked as incorrect.

The questionnaire employed in this study was a simplified tool designed to assess patient knowledge about azithromycin. Similar questionnaires focused on medication knowledge have been developed by other researchers, including Vitalina Rhozenfeld et al. [12], Mahendra et al. [13], Gangwar et al. [14], and others.

Scores were categorized as follows: good knowledge: >70%, average knowledge: 50–70%, and poor knowledge: <50%. These correct responses were developed in consultation with clinical pharmacists, and were aligned with established pharmacological references, including the Egyptian Drug Index, British National Formulary (BNF), and World Health Organization (WHO) guidelines. Additionally, the medication leaflet enclosed in the azithromycin packaging was also considered a reference.

The questionnaire also collected demographic data (e.g., age, gender, education, and occupation) and main information sources about azithromycin (e.g., doctors, pharmacists, or the internet). The study sample was stratified into upper- and lower-income groups for further analysis.

### Validation of the questionnaire

The validity of the questionnaire was established through expert review by three clinical pharmacists, who recommended minor revisions to improve clarity and relevance. Face validity was assessed through pre-testing with 10 participants, which led to additional refinements. Test-retest reliability was evaluated by administering the questionnaire to 15 participants on two occasions, separated by a two-week interval. Internal consistency was assessed using Cronbach’s alpha, which yielded a value of 0.78, indicating acceptable reliability. These findings support the questionnaire as a consistent and reliable instrument for evaluating patient knowledge about azithromycin.

### Data analysis

Data analysis was performed using SPSS version 26. Descriptive statistics, including mean ± standard deviation for continuous variables and frequencies with percentages for categorical variables, were used to summarize demographic characteristics, azithromycin usage patterns, and knowledge levels. The Kolmogorov-Smirnov test was used to assess the normality of continuous variables, including age and PKS. For comparisons between two groups, the independent samples t-test was used. For comparisons among three or more groups, one-way ANOVA was applied. When a statistically significant difference was found using ANOVA, post hoc analysis was conducted to determine which specific groups differed from each other. Odds ratios (OR) with 95% confidence intervals (CI) were calculated to identify predictors of average or good patient knowledge. A *p*-value of < 0.05 was considered statistically significant.

## Results

A total of 487 potential participants were approached for recruitment. Of these, 432 met the eligibility criteria and were invited to participate in the study. Among the eligible individuals, 418 provided informed consent and completed the questionnaire, resulting in a response rate of 96.76%. The remaining 14 eligible participants declined participation, citing reasons such as lack of time, disinterest, or unwillingness to disclose medication-related information. The sociodemographic characteristics of the study participants are presented in Table 1.

**Table 1:** Demographic characteristics of the study sample

Characteristics		
**Age/year**		
Mean ± SD	35.27 ± 11.08	
**Age group (years)**,** n (%)**		
< 40 years	316	(75.60)
40–60 years	92	(22.010
> 60 years	10	(2.39)
**Gender**,** n (%)**		
Male/Female	158/260	(37.80)/ (62.20)
**Marital status**,** n (%)**		
Married	342	(81.82)
Live separate, divorced, or widowed	76	(18.18)
**Residence**,** n (%)**		
Urban / Rural	242 / 176	(57.89)/ (42.11)
**Educational level**,** n (%)**		
No formal education	29	(6.94)
School level	106	(25.36)
University level	283	(67.70)
**Smoking**,** n (%)**		
Non-smokers	375	(89.71)
Smokers	43	(10.29)
**Diabetes mellitus**,** n (%)**		
Yes / No	67 / 351	(16.03) / (83.97)

SD, standard deviation

The study sample, as detailed in Table 1, had a mean age of 35.27 years, with 75.60% of participants under the age of 40. Females comprised 62.20% of the sample. The majority of participants were married (81.82%), and the sample was slightly more urban (57.89%) than rural. Educational attainment was high, with 67.70% of participants holding a university degree, which may have a positive influence on medication knowledge. Most participants were non-smokers (89.71%), and 16.03% had diabetes mellitus.

Table 2 highlights key aspects of azithromycin dosage regimens among the study participants. Physicians prescribed the antibiotic for 62.20% of participants, while 29.67% obtained it from pharmacists OTC, and 8.13% self-medicated. These findings emphasize the important role of healthcare professionals in guiding appropriate antibiotic use. Similarly, physicians (62.20%) and pharmacists (29.67%) were the primary sources of drug information, with minimal reliance on the internet or social networks. Most participants (93.78%) reported taking azithromycin at a dosage of 500 mg per day, with 91.87% administering it every 24 h. The most commonly reported treatment durations were 3 days (44.98%) and 5 days (51.20%).

**Table 2:** Azithromycin dosage regimens in the study sample

Dosage regimen characteristics	*n*	%
**Treatment prescriber**		
Self-medication	34	8.13
Physician	260	62.20
Pharmacist (OTC)	124	29.67
**Main sources of the drug information**		
Physician	260	62.20
Pharmacist	124	29.67
Internet/Online health websites	13	3.10
Personal and social networks	17	4.07
Educational materials	4	0.96
**Treatment dosage**		
500 mg/day	392	93.78
1000 mg/day	26	6.22
**Treatment frequency**		
Every 12 h	34	8.13
Every 24 h	384	91.87
**Treatment duration**		
3 Days	188	44.98
5 Days	214	51.20
*Other	16	3.82

*Other means the duration did not align with the standard 3-day or 5-day regimens.

The data in Table 3 reveal varying levels of patient awareness regarding the potential side effects of azithromycin. Gastrointestinal issues were the most commonly expected, with 31.82% of participants mentioning nausea or vomiting, 27.27% diarrhea, and 22.97% abdominal discomfort. In contrast, the expectation of central nervous system side effects, such as dizziness, was low (2.39%), although 27.99% of participants expected headache as a potential side effect. Cardiovascular side effects, such as palpitations, were anticipated by only 3.83%. Skin-related reactions, such as allergy, were mentioned by 8.37%, while 26.08% expected fatigue. Overall, participants were most familiar with common gastrointestinal side effects, highlighting the need for more comprehensive patient education on azithromycin’s full range of potential adverse effects.

**Table 3:** Patient knowledge about the potential side effects of azithromycin

	*n*	%
**Gastrointestinal**		
Diarrhea	114	27.27
Nausea or vomiting	133	31.82
Abdominal pain or discomfort	96	22.97
**Central nervous system**		
Headache	117	27.99
Dizziness	10	2.39
**Cardiovascular system**		
Palpitation	16	3.83
**Skin**		
Skin allergy or rash	35	8.37
**General**		
Fatigue	109	26.08

The table also indicates that some participants demonstrated misconceptions about azithromycin’s adverse effects. A number of them cited symptoms that are rarely reported and not commonly associated with azithromycin use, suggesting the need for clearer patient counseling and improved access to reliable drug information.

The results in Table 4 reveal significant relationships between PKS regarding azithromycin and various sociodemographic factors. Age, gender, smoking status, and diabetes mellitus did not significantly influence knowledge levels. However, married individuals scored higher (63.5%) than single, divorced, or widowed participants (58.1%, *p* = 0.002), suggesting that marital relationships may contribute to enhanced health knowledge. Urban residents also demonstrated higher scores (64.8%) compared to rural residents (59.4%, *p* < 0.001), indicating disparities in access to health information. Educational level emerged as a strong predictor, with university-educated participants scoring significantly higher (67.5%) than those with a school-level education (51.8%) or no formal education (53.1%, *p* < 0.001). Similarly, individuals with higher socioeconomic status achieved better knowledge scores (69% vs. 56%, *p* < 0.001).

**Table 4:** Relationship between patient knowledge scores (PKS) about azithromycin and sociodemographic characteristics

Sociodemographic characteristics			*n*	%	PKS M (SD) %	*P*
**Age group (years)**						
< 40 years			316	75.60	62.6 (13.1)	
40–60 years			92	22.01	62.5 (14.3)	0.8
> 60 years			10	2.39	60 (12)	
**Gender**						
Male			158	37.80	63.8 (12.9)	0.12
Female			260	62.20	61.7 (13.5)	
**Marital status**						
Single, divorced or widowed, n (%)			76	18.18	58.1 (15.9)	**0.002**
Married, n (%)			342	81.82	63.5 (12.5)	
**Residence**						
Urban			242	57.89	64.8 (11.4)	**< 0.001**
Rural			176	42.11	59.4 (15)	
**Educational level**						
No formal education			29	6.94	53.1 (16.2)	
School level			106	25.36	51.8 (9.6)	**< 0.001**
University level			283	67.70	67.5 (11.1)	
P1	0.56	P2		**< 0.001**	P3	**< 0.001**
**Socioeconomic status**						
Lower half (lower 50%) of income			209	50	56 (11.7)	**< 0.001**
Upper half (upper 50%) of income			209	50	69 (11.5)	
**Smoking**						
Smokers			43	10.29	64.2 (13.7)	0.38
Non-smokers			375	89.71	62.3 (13.2)	
**Diabetes mellitus**						
Diabetic			67	16.03	61.1 (12.7)	0.36
Non-diabetic			351	83.97	62.8 (13.4)	

The independent samples t-test was used for comparisons between two groups. One-way ANOVA was applied for comparisons among three or more groups, and post hoc analysis was conducted to determine which specific groups differed from each other. The *p*-value was considered significant if it was less than 0.05. The comparisons are as follows: p1 represents the comparison between the no formal education group and the school-level group; p2 represents the comparison between the no formal education group and the university-level group; and p3 represents the comparison between the school-level group and the university-level group.

**Fig. 3:**
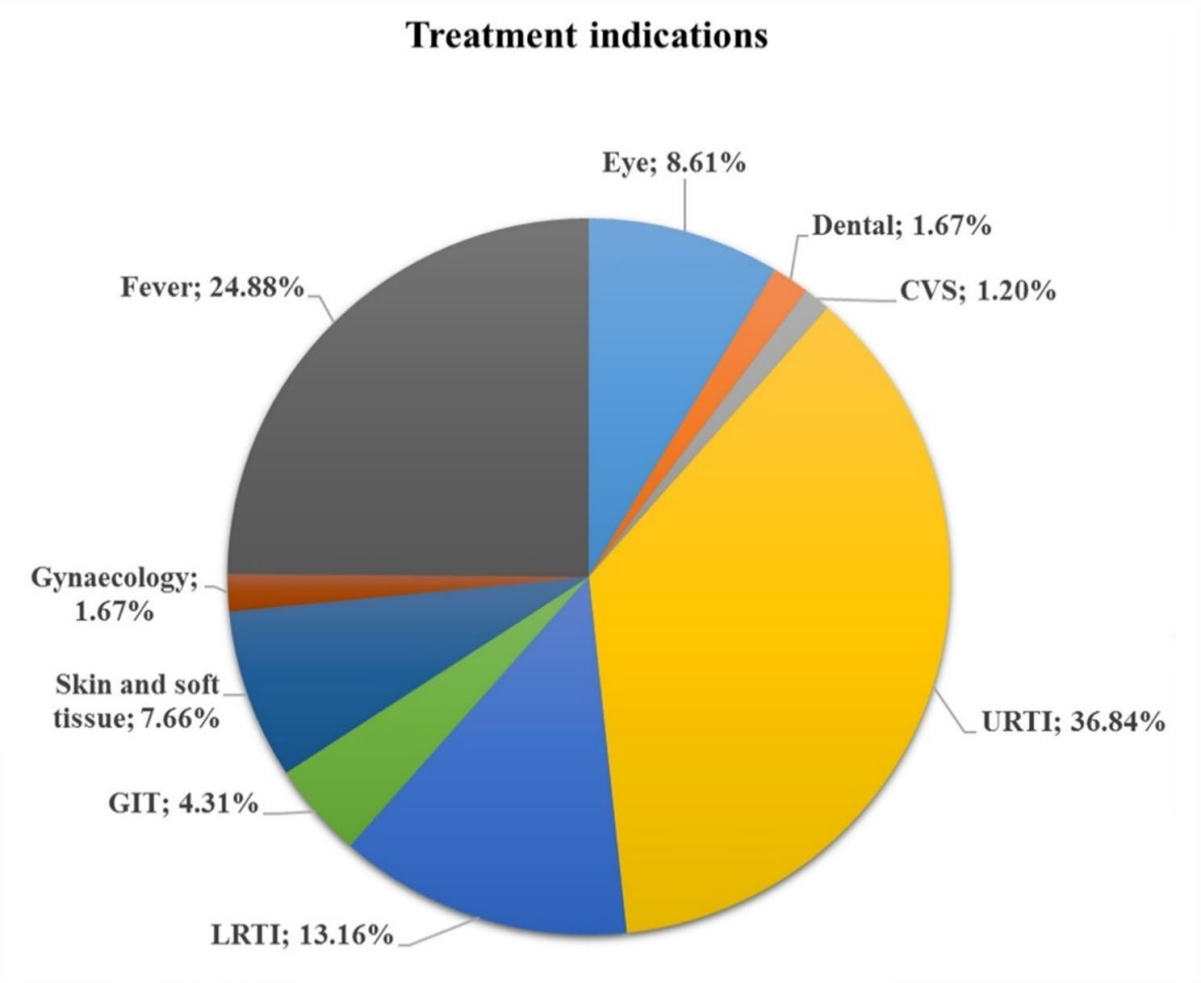
Indications for azithromycin use in the study sample. Abbreviations: CVS (cardiovascular system), URTI (upper respiratory tract infections), LRTI (lower respiratory tract infections), and GIT (gastrointestinal tract).

Figure 3 illustrates the various indications for azithromycin use. When asked about the reason for using the medication, participants most frequently reported upper respiratory tract infections (URTI) (36.84%), followed by fever (24.88%) and lower respiratory tract infections (LRTI) (13.16%), highlighting azithromycin’s primary role in managing respiratory infections (Fig. 3). Other reported indications included eye infections (8.61%), skin and soft tissue infections (7.66%), and gastrointestinal tract (GIT) infections (4.31%). Less commonly cited indications were dental infections (1.67%), gynecological conditions (1.67%), and cardiovascular system (CVS) infections (1.20%), suggesting limited perceived use for these conditions.

**Fig. 4:**
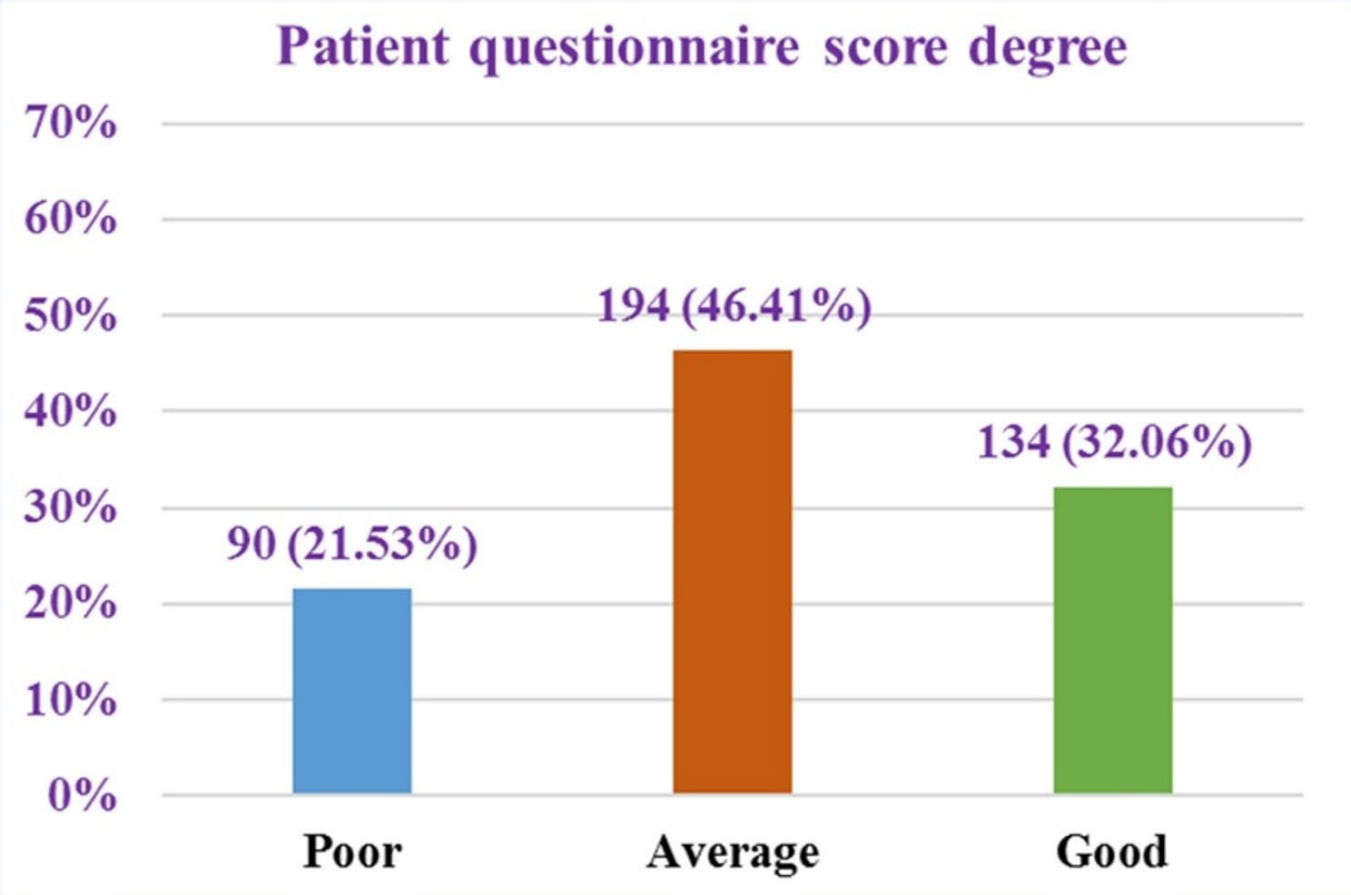
Distribution of patient knowledge scores in the study sample

Figure 4 shows that 90 participants (21.53%) had a poor level of knowledge, while 194 participants (46.41%) demonstrated an average level of knowledge. In contrast, 134 participants (32.06%) exhibited a good level of knowledge (Fig. 4).

**Fig. 5:**
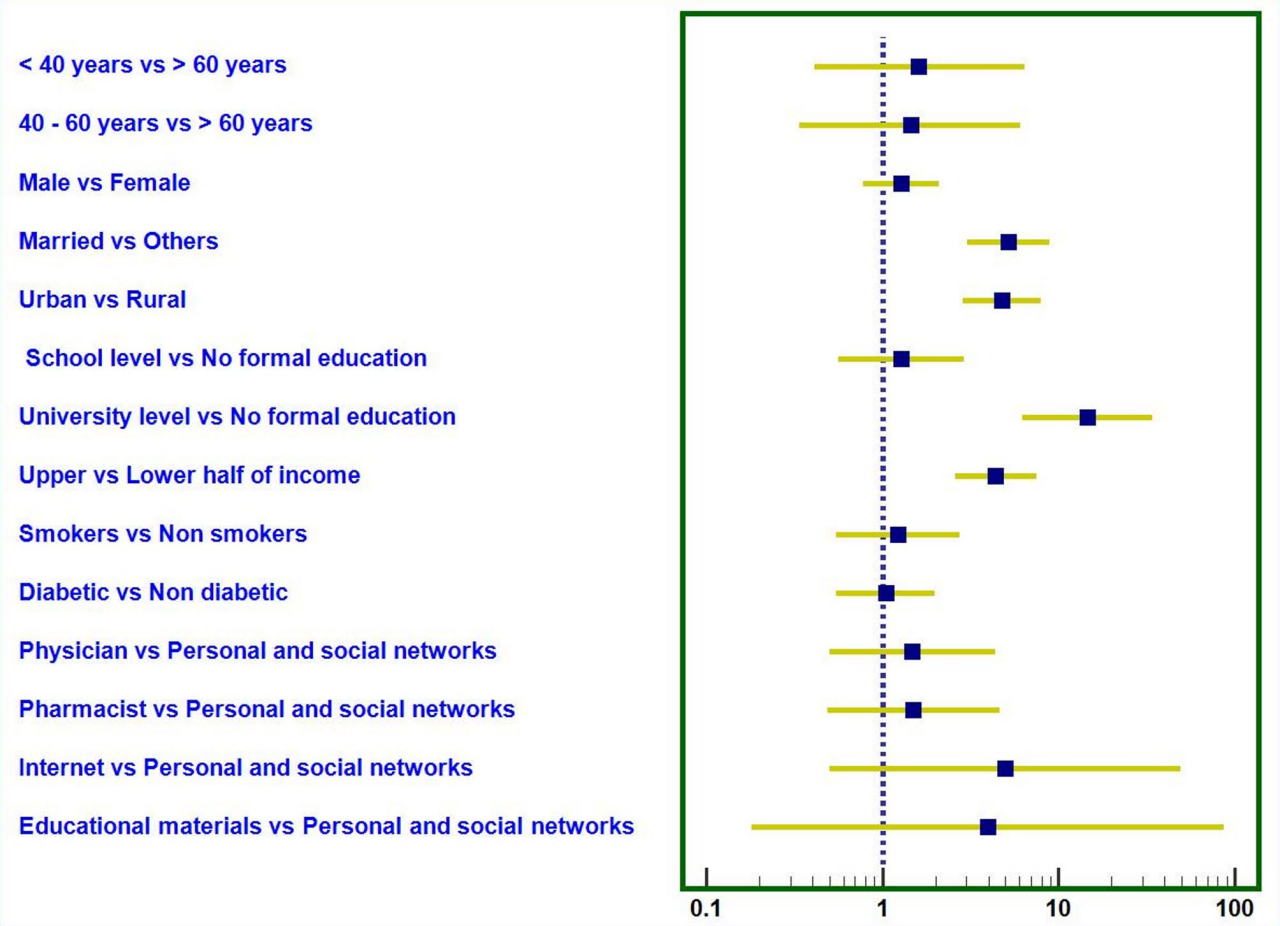
Predictors of average or good patient medication knowledge about azithromycin. Factors in order: Age group, gender, occupation, marital status, residence, education, income, smoking, diabetes mellitus, main source of drug information

Figure 5 shows that age, gender, smoking status, diabetes mellitus, and the main source of drug information had no significant impact on patient knowledge. However, married individuals and urban residents had higher odds of demonstrating better knowledge, indicating disparities in access to health information. Additionally, university education and higher socioeconomic status were strong predictors of improved knowledge (Fig. 5).

## Discussion

This study found that 75.60% of participants were under 40, 22.01% were 40–60, and 2.39% were over 60, with more urban than rural residents. Urban conditions like overcrowding, noise, pollution, and heat islands increase chronic diseases such as diabetes, asthma, cardiovascular issues, and infections, leading to higher antibacterial use [15]

In the current study self-medication with azithromycin, reported by 8.13% of study participants, involves improper antibiotic use, such as using others’ prescriptions, leftovers, or without medical consultation [16]. Globally, its prevalence ranges from 32.5–81.5% [17], posing a substantial public health challenge in both industrialized and developing countries [18]. This practice can lead to incorrect medication choices, inappropriate dosages, delayed medical care, adverse reactions, and increased antibacterial resistance [19]

The present study revealed that, 62.2% of prescriptions came from physicians, the most reliable method for accurate diagnosis and treatment. Despite being primarily prescription-only, azithromycin is available OTC in some pharmacies [20]. The current study evaluated patient knowledge, considering prescriber communication, pharmacist counseling, and self-learning, regardless of how the medication was obtained

The standard adult dose of azithromycin is 500 mg daily, but higher doses like 1000 mg daily are recommended for specific infections such as genital ulcer disease, urethritis, and cervicitis [21]. A 1000 mg single-dose regimen is advised for certain sexually transmitted infections like Chlamydia trachomatis, acute bacterial sinusitis, or community-acquired pneumonia to quickly achieve therapeutic levels [22–24]. Physicians typically prescribe 500 mg daily, adjusting based on the patient’s condition [25]

Potential side effects of Azithromycin included nausea, vomiting, diarrhea, abdominal discomfort, headache, and allergic reactions [26, 27]. However, some participants exhibited misconceptions, citing symptoms such as dizziness, palpitations, and fatigue as potential adverse effects of azithromycin, despite these being less commonly or rarely associated with the drug

In this study, azithromycin was primarily used for upper respiratory tract infections (URTIs), with fever and respiratory symptoms being the main reasons for self-medication with antibiotics [16]. Beyond URTIs, azithromycin is also effective for asthma, bronchiolitis, COPD, cystic fibrosis, enteric infections, sexually transmitted infections, and periodontal infections [28]

The present study found no significant differences in PKS about azithromycin across age groups, likely due to widespread access to medical information online and on social media. A separate study supported this, finding no correlation between knowledge, habits, and demographics [29]. However, another study noted that younger individuals were more likely to use the internet for health information and self-medicate [16]. Additionally, age (≥ 65 years) was linked to lower medication knowledge scores in other research [30].

The current study found no significant difference in PKS about azithromycin between males and females, consistent with a study showing no gender-based differences in knowledge, attitudes, and habits regarding drug-food interactions [29]. However, another study suggested females had higher medication knowledge scores, influenced by higher education, younger age, and shorter medication use duration [30].

Marital status was significantly linked to higher PKS about azithromycin in this study, likely due to collaborative health decision-making and supportive dynamics in marriages, which improve healthcare access and outcomes. Other studies similarly noted that married individuals, urban residents, and those with higher education were more likely to adhere to prescribed medications and less likely to miss doses [31, 32].

Urban residents in the current study demonstrated significantly higher PKS compared to rural residents. This disparity may be attributed to improved access to healthcare facilities, community pharmacies, and internet-based medical information in urban areas. Supporting this, a study conducted in China reported that rural residents exhibited less favorable attitudes toward medication use [33].

Participants with university-level education achieved significantly higher PKS compared to those with lower educational attainment. This result is consistent with earlier research demonstrating that individuals with high school degrees or higher had significantly better medication adherence and knowledge than those with no formal education (*p* < 0.001) [30]. Additionally, another study identified male gender and lower education levels as key contributors to inadequate drug knowledge [33].

The current study found that participants with higher incomes had better medication knowledge than those with lower incomes, likely due to greater access to educational resources and professional guidance. This highlights how socioeconomic status affects health literacy and medication understanding, leading to disparities in healthcare quality and treatment adherence across income groups [34].

### Limitations

This study has limitations including its cross-sectional design, which only captured patient knowledge at a single point after obtaining azithromycin, limiting insights into knowledge changes over time. It was conducted in specific community pharmacies in one region, reducing generalizability. The brief questionnaire, designed for engagement, may have limited data depth. Participants’ responses may reflect misconceptions rather than accurate knowledge, suggesting a need for more validated questionnaires to distinguish factual understanding from assumptions. Future longitudinal studies are recommended.

## Conclusion

This study highlights significant gaps in patients’ knowledge of azithromycin, with nearly half showing average understanding and many demonstrating poor knowledge. Higher knowledge was linked to being married, living in urban areas, having a university education, and higher socioeconomic status. Targeted education is needed for rural residents, those with lower education, and limited financial resources. Enhanced public understanding through pharmacist counseling and healthcare provider communication can improve patient outcomes and combat antibiotic resistance.

## Supplementary Information

Below is the link to the electronic supplementary material.

Supplementary Material 1

## Data Availability

The datasets used and/or analysed during the current study are available from the corresponding author on reasonable request.

## Abbreviations

AMR: Antimicrobial resistance
COPD: Chronic obstructive pulmonary disease
CVS: Cardiovascular system
GIT: Gastrointestinal tract
LRTI: Lower respiratory tract infections
PKS: Patient Knowledge Score
URTI: Upper respiratory tract infections
