# Supplementary material for: Assessing patients’ knowledge immediately after obtaining the antibacterial drug azithromycin: a community pharmacy-based cross-sectional study
Source: BMC Res Notes. 2025 Aug 18;18:358. doi: 10.1186/s13104-025-07447-1 (PMC12359916; doi:10.1186/s13104-025-07447-1)
Supplement: Supplementary file 1 — Supplementary Material 1 [file 13104_2025_7447_MOESM1_ESM.docx]

**Supplementary File: Patient Knowledge Assessment Questionnaire on Azithromycin**

**Introduction**

This questionnaire was developed for the study **"** **Assessing Patients’ Knowledge Immediately After Obtaining the Antibacterial Drug Azithromycin: A Community Pharmacy-Based Cross-Sectional Study"** It was used to evaluate patient knowledge regarding azithromycin through structured, face-to-face interviews conducted by pharmacists.

**Section 1: Demographic and Clinical Information**

1. **Age:** ________ years
2. **Gender:** ☐ Male ☐ Female
3. **Marital Status:** ☐ Single ☐ Married ☐ Divorced ☐ Widowed
4. **Educational Level:**
   ☐ No formal education ☐ Primary ☐ Secondary ☐ University
5. **Occupation:** _______________
6. **Residence:** ☐ Urban ☐ Rural
7. **Income Level:** ☐ Upper ☐ Lower
8. **Smoking Status:** ☐ Smoker ☐ Non-Smoker
9. **Do you have diabetes mellitus?** ☐ Yes ☐ No
10. **Have you used azithromycin before?** ☐ Yes ☐ No
11. **Who prescribed this treatment for you?**
    ☐ Self-medication ☐ Physician ☐ Pharmacist
12. **What is your primary source of information about azithromycin?**
    ☐ Physician ☐ Pharmacist ☐ Internet/Online health websites ☐ Personal and social networks ☐ Educational materials

**Section 2: Knowledge Assessment on Azithromycin**

**(Each question is scored as follows: Correct = 20%, Partially Correct = 10%, Incorrect/Don't Know = 0%)**

1. **What is the name of your dispensed medication?**
   *Response:* _______________
2. **What is the reason for taking this dispensed medication?**
   *Response:* _______________
3. **What is the dosage of your dispensed medication?**
   ☐ 500 mg/day
   ☐ 1000 mg/day
   ☐ Other: _________
   ☐ I don’t know
4. **How often should you take this dispensed medication?**
   ☐ Every 12 hours
   ☐ Every 24 hours
   ☐ Other: _________
   ☐ I don’t know
5. **For how many days should you take this dispensed medication?**
   ☐ 3 Days
   ☐ 5 Days
   ☐ Other: _________
   ☐ I don’t know
6. **What are the possible side effects of this dispensed medication?**
   *(Each correct response is awarded 5%, up to a maximum of 20%)*
   *Response:* _______________

**Scoring and Interpretation**

- **Good Knowledge:** >70%
- **Average Knowledge:** 50%–70%
- **Poor Knowledge:** <50%

**Instructions for Interviewers**

- Ensure that all questions are asked in a clear and neutral manner.
- Do not provide hints or correct participants’ answers.
- Record responses exactly as provided by participants.
- Clarify any ambiguities in responses but avoid leading questions.
